# First person – James Fabrizio

**DOI:** 10.1242/bio.051151

**Published:** 2020-02-26

## Abstract

First Person is a series of interviews with the first authors of a selection of papers published in Biology Open (BiO). James Fabrizio is first author on ‘ Tubulin-binding cofactor E-like (TBCEL), the protein product of the *mulet* gene, is required in the germline for the regulation of inter-flagellar microtubule dynamics during spermatid individualization’, published in BiO. James is a Professor of Biology/Jean Ames-DeNunzio Endowed Chair of Faculty Excellence at the College of Mount Saint Vincent, USA, investigating the genetics of spermatid individualization in *Drosophila melanogaster*.


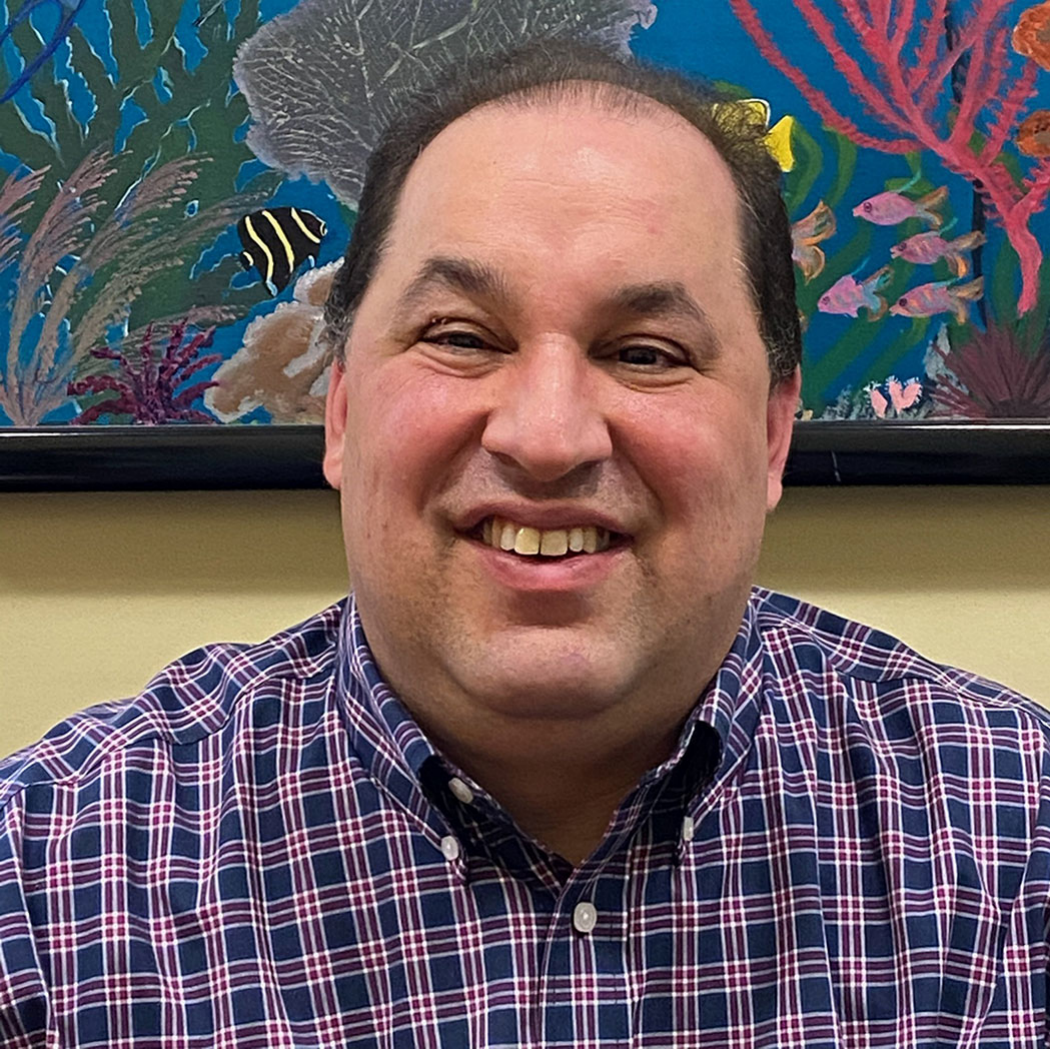


**James Fabrizio**

**What is your scientific background and the general focus of your lab?**

I have always studied sperm development in *Drosophila*! I was first introduced to this amazing process as a graduate student at St John's University, where I used genetics and cell biology to study spermatid individualization. Later, as a post-doctoral fellow at the University of Pennsylvania, I became more of a Developmental Biologist, studying the role of soma-to-germline communication during spermatocyte development. Since I joined the faculty at the College of Mount Saint Vincent, I have returned to spermatid individualization. While I no longer study spermatocytes, I still draw upon the wealth of knowledge and new ways of thinking provided to me by my post-doctoral training to study post-meiotic spermatogenesis. Currently, my lab is trying to understand the role that cytoplasmic microtubules play in spermatid individualization. Moreover, since the College of Mount Saint Vincent is a teaching college dedicated primarily to undergraduate education, my lab also seeks to expose undergraduates to original research. The thrill of research and discovery combined with the joy of learning, teaching and mentorship has always made our lab, the ‘FabLab’, a very special place on campus.

**How would you explain the main findings of your paper to non-scientific family and friends?**

Sperm development in all animals, including flies and mammals, occurs within a syncytium, or common cytoplasm. In other words, as sperm multiply and mature, they remain connected to each other by cytoplasmic bridges so that resources may be shared in a common ‘sac’. Only at the very end of spermatogenesis are individual sperm cells resolved, or ‘shrink-wrapped’, from this syncytium in a process known as sperm individualization. We first realized the *mulet* gene was important for the individualization process when we looked at testes dissected from flies that were mutant for *mulet*; the males were sterile, the spermatids were not individualized, and the individualization machinery was severely discoordinated. In this paper, we show that we can also cause this *mulet* defect by knocking down expression of the *mulet* gene specifically in the developing sperm, showing that *mulet* is required in the male sex cells themselves for individualization. In addition, when we drive expression of the normal *mulet* gene in the mutant testes, sort of like gene therapy for flies, spermatid individualization is fixed along with male fertility, showing that it is indeed *mulet* that is required for sperm development. That said, we are beginning to realize that the sperm defect in *mulet* mutant males is probably not the result of a problem with the individualization machinery itself. Indeed, the *mulet* gene encodes a protein that remodels the sperm sac before individualization so that the shrink-wrapping process can occur in a coordinated fashion. Specifically, *mulet* is needed for the removal of a set of cellular cables, or microtubules, from between the sperm tails; we know this because electron microscopy reveals the presence of excess microtubules around each sperm tail in *mulet* mutant testes. Normally, the individualization machinery assembles at the sperm heads and travels down the sperm tails, shrink-wrapping each spermatid into a membrane as it moves along the sperm tails. In *mulet* mutant testes, excess microtubules remain between the sperm tails and appear to ‘derail’ the individualization machinery, thus producing a discoordinated individualization machine that is characteristic of the *mulet* mutation.

**What are the potential implications of these results for your field of research?**

A failure to individualize sperm results in sperm cells with excess cytoplasm attached to their tails, which makes it very difficult for the sperm to swim. These ‘cytoplasmic droplet’ sperm are found in most infertile men. Indeed, a failure of individualization is responsible for the majority of human male infertility cases. Given the genetic similarity between flies and humans, we hope to uncover genes responsible for sperm individualization in *Drosophila* so that our results may be applied to the human system. Our lab studies sperm development and male infertility. The leading cause of male infertility is a failure at the very end of spermatogenesis. *Drosophila* is an exceptional organism for biological research; it is easy to maintain in the laboratory, possesses strong mutational genetics, a sequenced genome, and ∼65% genetic homology to human beings. *Drosophila* also possesses gigantic sperm cells – almost 2 mm long! Thus, we can easily use *Drosophila* to study spermatogenesis, and due to the genetic similarity to humans, the results we obtain will apply directly to human spermatogenesis and fertility.

“Failure of individualization is responsible for the majority of human male infertility cases.”

**Figure:**
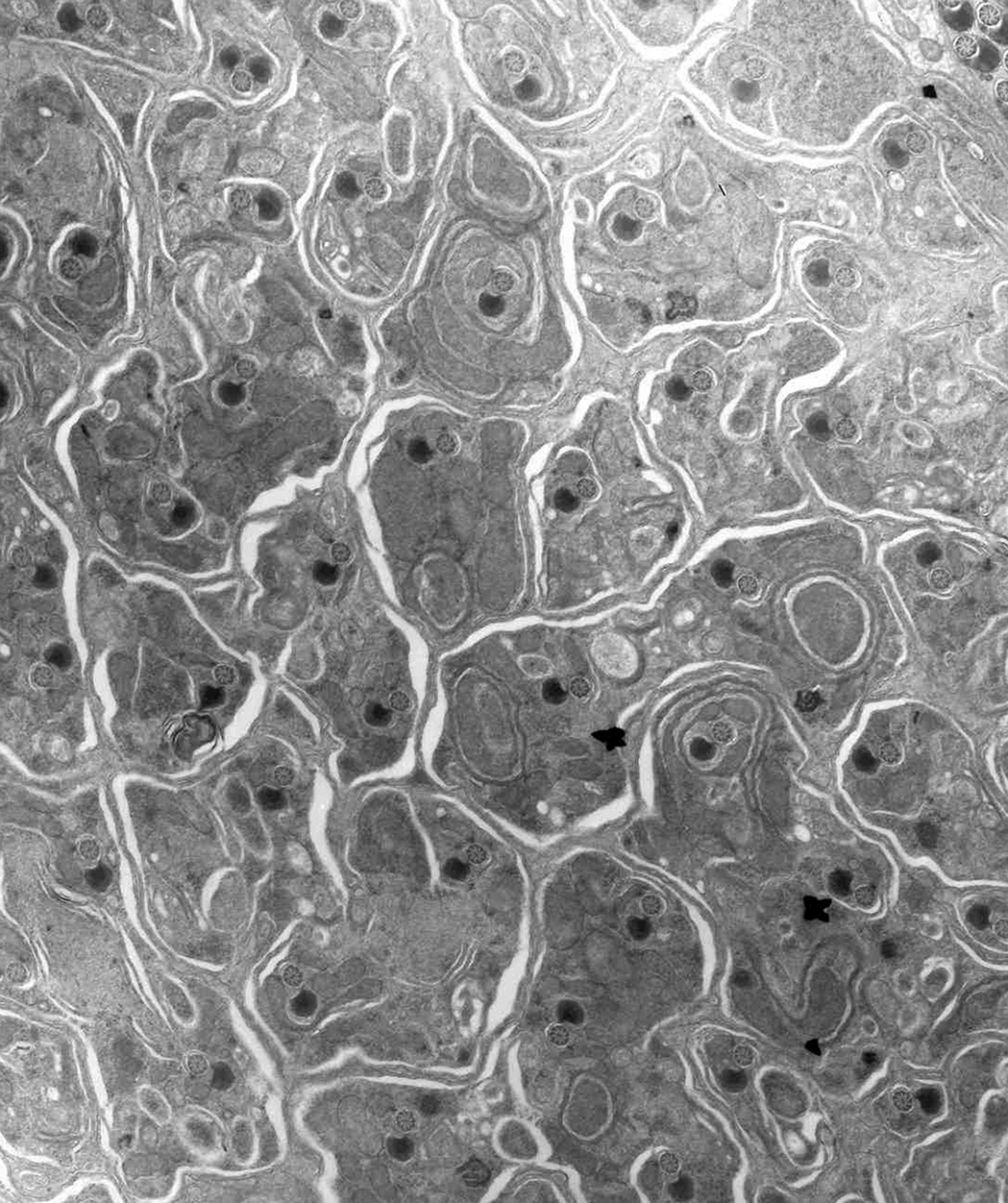
**This is a rare section through the membrane portion of the individualization complex, taken by my co-author Janet Rollins, viewed by transmission electron microscopy.** Notice the whorls of membrane spiralling around each axoneme as individualization proceeds.

**What has surprised you the most while conducting your research?**

The most interesting (and puzzling) aspect of the work has been the inverse correlation between the severity of both the *mulet* mutant alleles and RNAi-mediated *mulet* knockdown and the severity of the individualization phenotype. In other words, we see a weak individualization phenotype when *mulet* expression is knocked down severely but a severe individualization phenotype when *mulet* is knocked down modestly! We believe the explanation for this resides in the role of the *mulet* gene product, Tubulin-binding cofactor E-like (TBCEL) in removing a population of cytoplasmic microtubules from between the sperm tails prior to individualization so that the individualization complex can move unimpeded along the flagella. Thus, in cases of moderate TBCEL knockdown, we believe that these cytoplasmic microtubules are not completely degraded, resulting in microtubules fragments between the flagella that derail the individualization machinery, causing a severe disruption in the structure of the individualization complex. In contrast, in the case of severe TBCEL knockdown, cytoplasmic microtubules may be left intact, providing the individualization complex with a continuous, alternate route to the tips of the sperm tails.

**What, in your opinion, are some of the greatest achievements in your field and how has this influenced your research?**

I suppose for me it all began with the work of Tokuyasu, Peacock and Hardy in 1972, who first beautifully characterized *Drosophila* spermatid individualization in *Drosophila* using the electron microscope. Many years later, Steve Wasserman and Steve DiNardo published a paper (Castrillon et al., 1993) that essentially provided a gold mine of genes that affect spermatid individualization; *mulet* came from that screen! These two papers together set the stage for my PhD thesis and my first paper on spermatid individualization (Fabrizio et al., 1998), where we characterized the process by analyzing spermatid individualization mutants using a fluorescence assay for the individualization complex. Certainly, the work from Kathryn Miller's lab, which revealed the molecular components of the investment cones of the IC, and Eli Arama's lab, which revealed much of the mechanism behind spermatid individualization, have been a constant source of inspiration for me. Finally, the use of the Gal4/UAS system in *Drosophila,* first for overexpression and rescue and more recently for driving RNAi, has been a critical technical advancement that made our work possible.

**What changes do you think could improve the professional lives of early-career scientists?**

I have been running a small undergraduate research lab for the past 17 years, so that world of big labs with big grants, graduate students and post-docs is far away for me. However, as a professor at a small liberal arts college who spends most of his time teaching and mentoring undergraduates, perhaps I can offer a unique perspective. Would a career in undergraduate education be an improvement in the professional lives of early-career scientists? For many, I think absolutely, yes. We in undergraduate education need the talents and passions of early-career scientists! Colleges are full of raw talent in the form of undergraduate students, who are just waiting to be inspired by passionate and dedicated scientists with a zeal for teaching and mentorship. We have the pleasure of ‘planting the seeds’ in these eager young minds, a pleasure that is very often lost in the education of graduate students. Moreover, while our career does require productive and engaged scholarship, our livelihood is not solely dependent on grant money and publications. This leaves room for the joys of teaching, learning and science without the enormous pressures of a high-powered research environment. So to return to the original question, I think it is important that early-career scientists get some teaching experience if they think they might one day consider a career in undergraduate education. And I believe a career in undergraduate education can itself be an improvement in a scientist's professional life.

“We in undergraduate education need the talents and passions of early-career scientists!”

**What's next for you?**

My next group of undergraduate scientists and I will begin to examine how the various components of the individualization complex are affected by the *mulet* mutation. Are the investment cones of these individualization complexes really normal? Is the massive machine that these cones are pushing along the sperm tails normal? We will examine the localization of various markers in the *mulet* mutant background to address these questions. If possible, we would also love to capture movies of the individualization process using spinning disk confocal microscopy, and perhaps use the technique to compare individualization dynamics between wild-type and *mulet* mutant testes. I also look forward to finding ways of integrating this research into the laboratory component of my Developmental Biology course in the fall!
